# Risk assessment in sepsis: a new prognostication rule by APACHE II score and serum soluble urokinase plasminogen activator receptor

**DOI:** 10.1186/cc11463

**Published:** 2012-08-08

**Authors:** Evangelos J Giamarellos-Bourboulis, Anna Norrby-Teglund, Vassiliki Mylona, Athina Savva, Iraklis Tsangaris, Ioanna Dimopoulou, Maria Mouktaroudi, Maria Raftogiannis, Marianna Georgitsi, Anna Linnér, George Adamis, Anastasia Antonopoulou, Efterpi Apostolidou, Michael Chrisofos, Chrisostomos Katsenos, Ioannis Koutelidakis, Katerina Kotzampassi, George Koratzanis, Marina Koupetori, Ioannis Kritselis, Korina Lymberopoulou, Konstantinos Mandragos, Androniki Marioli, Jonas Sundén-Cullberg, Anna Mega, Athanassios Prekates, Christina Routsi, Charalambos Gogos, Carl-Johan Treutiger, Apostolos Armaganidis, George Dimopoulos

**Affiliations:** 1 4th Department of Internal Medicine, University of Athens, Medical School, 12462 Athens, Greece; 2 Center for Infectious Medicine F59, Karolinska Institutet, 14186 Stockholm, Sweden; 3 2nd Department of Internal Medicine, 'Sismanogleion' Athens Hospital, 15126 Athens Greece; 4 2nd Department of Critical Care, University of Athens, Medical School, 12462 Athens, Greece; 5 1st Department of Internal Medicine, 'G. Gennimatas' Athens Hospital, 115 27 Athens, Greece; 6 Intensive Care Unit, Ptolemaida General Hospital, 50400, Ptolemaida, Greece; 7 2nd Department of Urology, University of Athens, Medical School, 15126 Athens, Greece; 8 Intensive Care Unit, 'Korgialeneion-Benakeion' Athens Hospital, 11526 Athens, Greece; 9 2nd Department of Surgery, University of Thessaloniki, Medical School, 54635 Thessaloniki, Greece; 10 1st Department of Propedeutic Surgery, University of Thessaloniki, Medical School, 54635 Thessaloniki, Greece; 11 1st Department of Internal Medicine, 'Thriassio' Elefsina General Hospital, 19600 Magoula, Greece; 12 Department of Surgery, Nafplion General Hospital, 21100 Nafplion, Greece; 13 Intensive Care Unit, 'Laikon' Athens General Hospital, 11527 Athens, Greece; 14 Intensive Care Unit, 'Tzaneion' Hospital of Piraeus, 18536 Piraeus, Greece; 15 1st Department of Critical Care, University of Athens, Medical School, 10676 Athens, Greece; 16 1st Department of Internal Medicine, University of Patras, Medical School, 26504 Rion, Greece

## Abstract

**Introduction:**

Early risk assessment is the mainstay of management of patients with sepsis. APACHE II is the gold standard prognostic stratification system. A prediction rule that aimed to improve prognostication by APACHE II with the application of serum suPAR (soluble urokinase plasminogen activator receptor) is developed.

**Methods:**

A prospective study cohort enrolled 1914 patients with sepsis including 62.2% with sepsis and 37.8% with severe sepsis/septic shock. Serum suPAR was measured in samples drawn after diagnosis by an enzyme-immunoabsorbent assay; in 367 patients sequential measurements were performed. After ROC analysis and multivariate logistic regression analysis a prediction rule for risk was developed. The rule was validated in a double-blind fashion by an independent confirmation cohort of 196 sepsis patients, predominantly severe sepsis/septic shock patients, from Sweden.

**Results:**

Serum suPAR remained stable within survivors and non-survivors for 10 days. Regression analysis showed that APACHE II ≥17 and suPAR ≥12 ng/ml were independently associated with unfavorable outcome. Four strata of risk were identified: i) APACHE II <17 and suPAR <12 ng/ml with mortality 5.5%; ii) APACHE II < 17 and suPAR ≥12 ng/ml with mortality 17.4%; iii) APACHE II ≥ 17 and suPAR <12 ng/ml with mortality 37.4%; and iv) APACHE II ≥17 and suPAR ≥12 ng/ml with mortality 51.7%. This prediction rule was confirmed by the Swedish cohort.

**Conclusions:**

A novel prediction rule with four levels of risk in sepsis based on APACHE II score and serum suPAR is proposed. Prognostication by this rule is confirmed by an independent cohort.

## Introduction

Severe sepsis and septic shock are among the leading causes of death worldwide. Their incidence is constantly increasing, and almost 1,500,000 cases of severe sepsis and septic shock occur annually in North America and another 1,500,000 cases in Europe. Despite early intervention with antimicrobials, fluid resuscitation, and management in intensive care units (ICUs), mortality remains high, often exceeding 30%. This can be explained, in part, by the coexistence of chronic health disorders and the increasing rate of antimicrobial resistance that complicates management [1].

The mainstay in the proper management of sepsis is early recognition of the patient at high risk for death. This is traditionally based on the application of severity scores and serum biomarkers. The most widely applied score is that of the Acute Physiology and Chronic Health Evaluation II (APACHE II). However, APACHE II has several limitations that can give a misleading score. For example, in the case of young patients with severe sepsis but without chronic organ failures, the APACHE II score may be relatively low despite the risk of an unfavorable outcome. In contrast, older septic patients with chronic organ failures may provide high APACHE II scores even when the risk for dying from sepsis is low [2].

Urokinase plasminogen activator receptor (uPAR) is embedded in the cell membranes of leukocytes. Its soluble counterpart, suPAR, has been reported as a marker of severity and unfavorable outcome in a variety of diseases ranging from certain types of cancer to infectious diseases [3-5]. Recent studies with limited numbers of patients with bacteremia or sepsis are most relevant to this study and suggest that suPAR may inform about the risk of death [6,7]. However, in these studies, suPAR is not superior to APACHE II.

The present study aimed to develop and evaluate a new prognostication score of the risk for death in sepsis by using suPAR to improve information provided by the APACHE II score. To this end, one prospective cohort of patients enrolled by 39 departments participating in the Hellenic Sepsis Study Group [8] was studied. Results were confirmed in a second independent cohort of sepsis patients prospectively enrolled in an ICU in Sweden.

## Materials and methods

### Study design

This prospective multicenter study was conducted in 39 departments across Greece from January 2008 to December 2010. The participating departments were 15 ICUs, 18 departments of internal medicine, two departments of pulmonary medicine, three departments of surgery, and one department of urology.

Sepsis patients admitted to the emergency department and sepsis patients presenting during hospitalization in the general ward or in the ICU were eligible. Written informed consent was provided by the patients or by first-degree relatives of patients unable to provide consent. The study protocol was approved by the ethics committees of the participating hospitals. Each patient was enrolled once.

Inclusion criteria were (a) age of at least 18 years; (b) diagnosis of sepsis, severe sepsis, or septic shock; (c) sepsis due to one of the following infections: community-acquired pneumonia (CAP), hospital-acquired pneumonia, ventilator-associated pneumonia, acute pyelonephritis, intra-abdominal infection, or primary bacteremia; and (d) blood sampling within 24 hours from the presentation of signs of sepsis. Six study sites (two ICUs, three departments of internal medicine, and one department of surgery) were selected to be representative of the total enrolment study sites, and agreed to repeat blood sampling on days 3, 7, and 10. First sampling was always done before the administration of any treatment. Exclusion criteria were HIV infection and neutropenia, which was defined as less than 1,000 neutrophils/mm^3^.

Patients were classified according to standard definitions of sepsis, severe sepsis, and septic shock [9]. Infections were defined according to standard definitions [10-14]. For each patient, a complete diagnostic work-up was performed. The work-up comprised history, a thorough physical examination, white blood cell count, blood biochemistry, arterial blood gas, blood cultures from peripheral veins and central lines, urine cultures, a chest x-ray, and chest and abdominal computed tomography if appropriate. If necessary, quantitative cultures of tracheobronchial secretions (TBSs) or bronchoalveolar lavage (BAL) were performed and evaluated as previously described [11,12]. Progress was recorded for 28 days. Clinical and demographic data were recorded in a Case Report Form (CRF). All CRFs were monitored by an independent monitor who was blinded to the study design.

### Blood sampling and laboratory procedure

Blood (3 mL) was sampled from every patient. In 367 patients, blood sampling was repeated on days 3, 7, and 10. Blood was collected into sterile and pyrogen- and anticoagulant-free tubes (Vacutainer; Becton Dickinson, Cockeysville, MD, USA). On the same day, tubes were transported by courier to the Laboratory of Immunology of Infectious Diseases of the 4th Department of Internal Medicine at ATTIKON University Hospital (Athens). Tubes were centrifuged, and serum was kept frozen at −70°C until assayed. suPAR was estimated in duplicate by an enzyme-linked immunosorbent assay (suPARnostic™; ViroGates, Lyngby, Denmark) in accordance with the instructions of the manufacturer; the lower detection limit was 1.1 ng/mL. All estimations were performed and reported by two technicians who were blinded to clinical information.

### Primary endpoint

The primary endpoint was to create an accurate prognostication score of death by combining APACHE II score and serum suPAR. The value of this prognostication score was confirmed in a second independent cohort.

### Confirmation cohort

The confirmation cohort consisted of pooled results from three prospective studies and involved patients who were at least 18 years old; who had sepsis, severe sepsis, or septic shock; and who were enrolled in three prospective studies of patients with sepsis at the ICU of Karolinska University Hospital in Huddinge, Sweden, during the period of 1998 to 2009. The majority were from the ICU, but five patients were from wards. Demographic characteristics for some of these patients have been published [15-17]. Sepsis, severe sepsis, and septic shock were defined according to the criteria proposed by the American College of Chest Physicians/Society of Critical Care Medicine [9]. Serum was collected from blood sampled within the first day from diagnosis and was stored at −70°C until analysis. Samples were transported to the same central lab for analysis of suPAR. The technicians did not have access to the clinical information for patients. Based on the results of APACHE II scores and suPAR, a prognostication score was created with which patients in this confirmation cohort were graded for severity. Final matching of prognostication scores and outcome of sepsis was done in Sweden. This ensured that confirmation was conducted in a double-blinded manner.

### Statistical analysis

Acute-phase suPAR in serum was not normally distributed as assessed by Kolmogorov-Smirnov statistics. Therefore, serum suPAR was expressed as median and range or 95% confidence intervals (CIs). Comparisons between survivors and non-survivors were done by using Mann-Whitney *U *test. Comparisons between sequential measurements were performed separately among survivors and non-survivors by using the Wilcoxon signed-rank test.

Comparisons of demographic characteristics were done by chi-squared test for qualitative characteristics; for quantitative characteristics with normal distribution, the Student *t *test was used for two groups and analysis of variance was used for more than two groups.

To create a prognostication rule, the following steps were followed: First, receiver operator curve (ROC) analysis was done with suPAR of day 1 and APACHE II score as independent variables to predict unfavorable outcome. Values of APACHE II score and suPAR with an ROC analysis specificity of above 70% were selected. The latter specificity cutoff was selected since it was considered of importance for risk assessment in sepsis [18]. Second, the importance of the selected cutoffs as independent predictors of unfavorable outcome was defined by step-wise Cox regression analysis. Disease severity, selected cutoffs, and presence of at least one underlying disease were included as independent variables in the equation. Underlying diseases were chronic obstructive pulmonary disorder, diabetes mellitus type 2, chronic renal disease, heart failure, solid tumor malignancy, and chronic intake of corticosteroids since these disease states are widely recognized to affect final outcome. Age, white blood cells, and values of blood gases were not included in the regression analysis, because they were factored into APACHE II. Hazard ratios and 95% CIs were assessed. Third, ROC analysis of the combination of suPAR and APACHE II score was also performed. Given Cook's method of analysis of the role of biomarkers as indexes of disease severity [19], it is highly probable that the ROC generated by the combination of APACHE II score and suPAR does not provide an area under the curve (AUC) superior to that of single APACHE II score or single suPAR. To this end, four strata of severity were generated by using the defined cutoffs of APACHE II score and suPAR. Odds ratios (ORs) and 95% CIs for risk prediction within each stratum were calculated by using Mantel and Haenszel statistics. Comparisons between ORs were done by using Breslow-Day test and Tarone test. Fourth, mortalities between strata were compared by using the chi-squared test and log-rank test. Fifth, comparisons of the risk strata between the study cohort and the confirmation cohort were done by using the chi-squared test. *P *values of below 0.05 were considered significant.

## Results

### Study cohort

**Figure 1:**
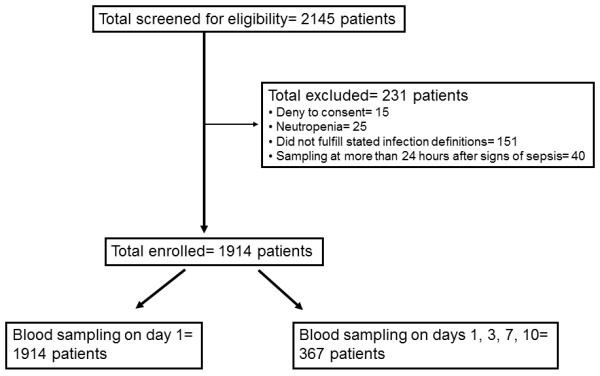
**Flowchart of enrolment of the Greek study cohort**.

A total of 1,914 patients were enrolled in the study cohort from a total of 2,145 patients screened for eligibility (Figure 1). All consecutively enrolled patients in the biobank of the Hellenic Sepsis Study Group during the period of January 2008 to December 2010 were included in the present study; 62.2% had sepsis and 37.8% had severe sepsis/septic shock. The most common causes of sepsis were acute pyelonephritis, intra-abdominal infections, and CAP; Gram-negative bacteria were the commonest isolated pathogens. More precisely, positive blood cultures for *Escherichia coli *were found in 113 patients (5.9%), for *Klebsiella pneumoniae *in 79 patients (4.1%), for *Pseudomonas aeruginosa *in 40 patients (2.1%), for *Acinetobacter baumannii *in 32 patients (1.7%), for other Gram-negative bacteria in 30 patients (1.5%), for *Staphylococcus aureus *in 21 patients (1.1%), and for *Enterococcus *spp in 15 patients (0.8%). Positive quantitative urine cultures for *E. coli *were found in 262 patients (13.7%), for *K. pneumoniae *in 44 patients (2.3%), for *P. aeruginosa *in 40 patients (2.1%), for other Gram-negative bacteria in 62 patients (3.3%), and for *Enterococcus *spp in 29 patients (1.5%). Positive quantitative TBS or BAL cultures for *A. baumannii *were found in 66 patients (3.6%), for *P. aeruginosa *in 36 patients (1.9%), for *K. pneumoniae *in 21 patients (1.1%), and for *S. aureus *in 16 patients (0.8%).

### Kinetics of suPAR

**Figure 2:**
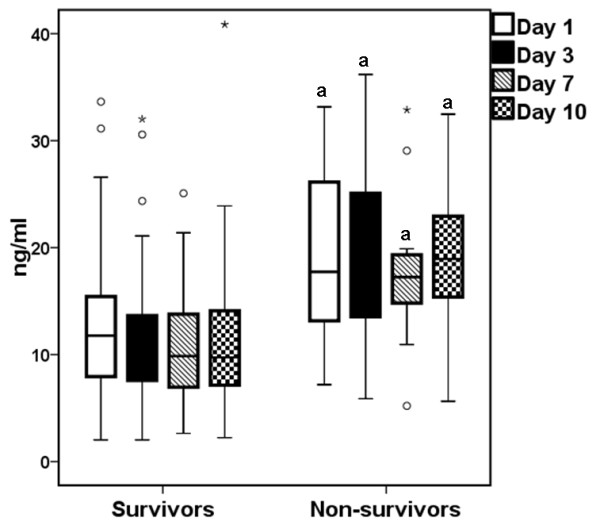
**Serum suPAR levels among 315 survivors and 52 non-survivors from sepsis over the course of 10 days**. Circles denote outliers, and asterisks above boxplots denote extremes. ^a^*P *< 0.0001 between survivors and non-survivors at the indicated day of sampling. suPAR, soluble urokinase plasminogen activator receptor.

Among the enrolled patients with sepsis, 1,495 patients survived and 419 died. Patients who died had significantly higher concentrations of suPAR. These patients had a median of 14.06 ng/mL (range of 2.92 to 69.84 ng/mL) in comparison with a median of 9.27 ng/mL (range of 1.14 to 65.00 ng/mL) in survivors (*P *< 0.0001). To define whether serum suPAR changes over time within survivors and non-survivors, sequential serum measurements were performed for 367 patients, 52 of whom died. At each time point, serum suPAR was significantly higher among non-survivors than among survivors (Figure 2). No significant differences were found after comparing sequential measurements of suPAR separately within survivors and within non-survivors.

### Receiver operator curve analysis

**Figure 3:**
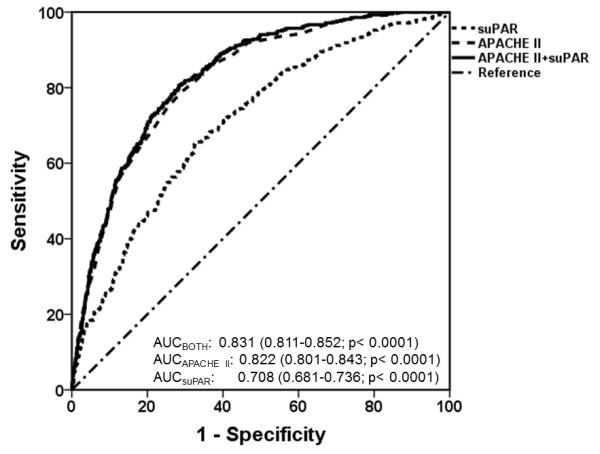
**Receiver operator curve (ROC) analyses of APACHE II score, serum suPAR, and their combination to define unfavorable outcome in a study cohort of 1,914 Greek patients**. Areas under curve (AUCs) and 95% confidence intervals are shown. APACHE II, Acute Physiology and Chronic Health Evaluation II; suPAR, soluble urokinase plasminogen activator receptor.

ROC analysis indicated that AUCs are much greater for APACHE II score than for suPAR (Figure 3). Coordinate points of ROCs define an APACHE II score of at least 17 as a cutoff and a specificity of greater than 70% to predict death. Similarly, suPAR of at least 12 ng/mL yields a specificity of greater than 70% to predict death.

### Regression analysis

Given that the AUC of the combination of APACHE II score and suPAR did not differ from that of a single APACHE II score, developing a new prognostication rule required that the above defined cutoffs for APACHE II score and suPAR be independently associated with unfavorable outcome. To investigate this, Cox logistic regression analysis was performed. In the equation, advent of death was set as the dependent variable. Two universally accepted conditions affecting final outcome − namely, the presence of severe sepsis/shock and the presence of at least one underlying disease − were also taken into consideration to try to decipher whether APACHE II score and suPAR may independently prognosticate for unfavorable outcome under the influence of these conditions. Analysis was done in a forward step-wise manner, and results are shown in Table 1. According to this analysis, serum suPAR of at least 12 ng/mL and APACHE II score of at least 17 retained an independent link with unfavorable outcome even when superimposed over the presence of severe sepsis/shock and the presence of underlying diseases. As a consequence, these two cutoffs may be safely used to build a prognostication rule for the assessment of unfavorable outcome in sepsis.

**Table 1:** Step-wise Cox regression analysis of factors related to unfavorable outcome in the study cohort of 1,914 Greek patients

Factor	Hazard ratio	95% CI	*P *value
Presence of severe sepsis/shock	1.46	1.32-1.61	<0.0001
At least one underlying disease^a^	1.78	1.39-2.28	<0.0001
APACHE II score ≥17	4.24	3.30-5.43	<0.0001
Serum suPAR ≥12 ng/mL	1.62	1.30-2.00	<0.0001

^a^Chronic obstructive pulmonary disorder, diabetes mellitus type 2, chronic renal disease, heart failure, solid tumor malignancy, and chronic intake of corticosteroids. APACHE II, Acute Physiology and Chronic Health Evaluation II; CI, confidence interval; suPAR, soluble urokinase plasminogen activator receptor.

It was then clearly defined that, among patients with an APACHE II score of less than 17 and among patients with an APACHE II score of at least 17, suPAR could significantly indicate those with high risk for death (Table 2). More precisely, OR for death with suPAR of at least 12 ng/mL among patients with an APACHE II score of less than 17 was 3.62; OR was 1.79 with suPAR of at least 12 ng/mL among patients with an APACHE II score of at least 17. The calculated ORs were significantly different (*P *of comparisons = 0.006 by the Breslow-Day test and *P *= 0.007 by the Tarone test), indicating that APACHE II score and suPAR were independent prognosticators of unfavorable outcome and should both be used in a prediction model.

**Table 2:** Validation of the new stratification scheme

APACHE II score	suPAR, ng/mL	Survivors, number (percentage)	Non-survivors, number (percentage)	*P *value	OR	95% CI
<17	<12	844 (94.5)	49 (5.5)	<0.0001	3.62	2.42-5.42
	≥12	276 (82.6)	58 (17.4)			
≥17	<12	184 (62.8)	109 (37.2)	<0.0001	1.79	1.32-2.44
	≥12	191 (48.5)	203 (51.5)			

APACHE II, Acute Physiology and Chronic Health Evaluation II; CI, confidence interval; OR, odds ratio; suPAR, soluble urokinase plasminogen activator receptor.

### Prognostication rule

**Figure 4:**
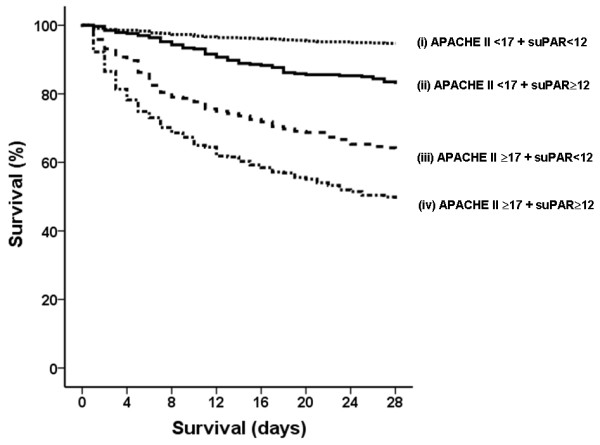
**Kaplan-Meier estimates of survival of patients enrolled in the study cohort stratified into four strata of severity by APACHE II score and serum suPAR**. Every curve differed significantly from the others. Log-rank tests of comparisons are stratum (i) versus stratum (ii) 43.93 (*P *< 0.0001), stratum (ii) versus stratum (iii) 33.72 (*P *< 0.0001), and stratum (iii) versus stratum (iv) 14.43 (*P *< 0.0001). APACHE II, Acute Physiology and Chronic Health Evaluation II; suPAR, soluble urokinase plasminogen activator receptor.

With the above cutoff values, four strata of sepsis severity were defined: (i) patients with an APACHE II score of less than 17 and a serum suPAR of less than 12 ng/mL, (ii) patients with an APACHE II score of less than 17 and a serum suPAR of at least 12 ng/mL, (iii) patients with an APACHE II score of at least 17 and a serum suPAR of less than 12 ng/mL, and (iv) patients with an APACHE II score of at least 17 and a serum suPAR of at least 12 ng/mL; 893, 334, 293, and 394 patients ended up in each stratum and had respective mortality rates of 5.5% (n = 49), 17.4% (n = 58), 37.4% (n = 109), and 51.5% (n = 203) (*P *< 0.0001 within the four defined strata; Figure 4).

This prognostication score corresponds to different grades of sepsis severity, so that more patients with severe sepsis and septic shock had score levels (iii) and (iv) than score levels (i) and (ii). As expected, coexisting illnesses were more common among patients of score levels (iii) and (iv) than score levels (i) and (ii) (Table 3). Sensitivity, specificity, positive predictive value, and negative predictive value of this new score to predict unfavorable outcome change in relation to the strata and are shown in Table 4.

**Table 3:** Characteristics of the 1,914 Greek patients stratified according to four degrees of severity by APACHE II score and serum suPAR

	APACHE II score <17 + suPAR <12 ng/mL	APACHE II score <17 + suPAR ≥12 ng/mL	APACHE II score ≥17 + suPAR <12 ng/mL	APACHE II score ≥17 + suPAR ≥12 ng/mL	*P *value
Male/female	480/413	162/172	170/123	216/178	0.014
Age in years, mean ± SD	59.4 ± 21.4	68.3 ± 18.7	73.3 ± 14.9	74.2 ± 13.7	<0.0001
Sepsis stage, number (percentage)					<0.0001
Sepsis	747 (83.6)	232 (67.4)	113 (38.6)	95 (24.1)	
Severe sepsis	105 (11.8)	68 (20.3)	105 (35.8)	134 (34.0)	
Septic shock	41 (4.6)	43 (12.9)	75 (25.6)	164 (41.6)	
Underlying infections, number (percentage)					<0.0001
CAP	169 (18.9)	55 (16.5)	93 (31.7)	87 (22.1)	
HAP	32 (3.6)	12 (3.6)	30 (10.2)	35 (8.9)	
VAP	29 (2.3)	15 (4.5)	30 (10.2)	48 (12.2)	
UTI	346 (38.7)	128 (38.3)	67 (22.9)	94 (23.9)	
IAI	260 (29.1)	80 (23.9)	35 (11.9)	61 (15.5)	
BSI	57 (6.4)	44 (13.2)	38 (12.9)	69 (17.5)	
Underlying conditions, number (percentage)					<0.0001
COPD	78 (8.7)	33 (9.9)	45 (15.4)	54 (13.7)	
DM2	143 (16.1)	90 (26.9)	74 (25.3)	123 (31.2)	
Heart failure	104 (11.6)	49 (14.7)	62 (21.2)	98 (24.8)	
CRD	35 (3.9)	29 (8.7)	27 (9.2)	85 (21.6)	
Intake of corticosteroids	27 (3.0)	8 (2.3)	16 (5.5)	22 (5.6)	
Malignancy	42 (4.7)	18 (5.4)	17 (5.8)	30 (7.6)	
Length of ICU stay^a ^in days, median (range)	19.5 (1-312)	20.0 (2-317)	22.5 (1-309)	14.5 (1-240)	0.196

^a^Refers only to patients hospitalized in an intensive care unit (ICU). APACHE II, Acute Physiology and Chronic Health Evaluation II; BSI, bloodstream infection; CAP, community-acquired pneumonia; COPD, chronic obstructive pulmonary disorder; CRD, chronic renal disease; DM2, diabetes mellitus type 2; HAP, hospital-acquired pneumonia; IAI, intrabdominal infection; SD, standard deviation; suPAR, soluble urokinase plasminogen activator receptor; UTI, urinary tract infection; VAP, ventilator-associated pneumonia.

**Table 4:** Characteristics of the proposed prognostication rule to predict unfavorable outcome according to the strata where every patient belongs

	Sensitivity, percentage	Specificity, percentage	PPV, percentage	NPV, percentage
	Patients belonging to level (ii), (iii), or (iv) of the prognostication rule			
Greek cohort	88.3	56.5	36.2	94.5
Swedish cohort	97.6	31.2	27.9	97.9
	Patients belonging to level (iii) or (iv) of the prognostication rule			
Greek cohort	74.4	74.9	45.4	91.3
Swedish cohort	83.3	40.9	27.7	90.0
	Patients belonging to level (iv) of the prognostication rule			
Greek cohort	48.4	87.2	51.2	85.8
Swedish cohort	52.4	72.1	66.2	84.7

NPV, negative predictive value; PPV, positive predictive value.

### Confirmation analysis

In a further test of the predictive value of this stratification scheme, an independent confirmatory sepsis cohort was used. This cohort included 196 sepsis patients - 108 males and 88 females (*P *= 0.652 compared with the Greek cohort) - enrolled in Sweden. As shown in Table 5, the Swedish cohort differed considerably from the Greek study cohort in many aspects, including the following:

**Table 5:** Characteristics of the 196 Swedish patients stratified according to four degrees of severity by APACHE II score and serum suPAR

	APACHE II <17 + suPAR <12 ng/mL	APACHE II <17 + suPAR ≥12 ng/mL	APACHE II ≥17 + suPAR <12 ng/mL	APACHE II ≥17 + suPAR ≥12 ng/mL	*P *value
Male/female	28/21	11/10	33/28	36/29	0.986
Age in years, mean ± SD	56.3 ± 16.9	54.3 ±19.1	64.2 ± 14.9	64.1 ± 13.0	<0.0001
Sepsis stage, number (percentage)					0.015
Sepsis	2 (4.1)	0 (0)	0 (0)	0 (0)	
Severe sepsis	11 (22.4)	10 (47.6)	11 (18.0)	11 (16.9)	
Septic shock	36 (73.5)	11 (52.3)	50 (82.0)	54 (83.1)	
Underlying infections, number (percentage)					0.008
CAP	10 (20.5)	4 (19.1)	12 (19.6)	13 (20.0)	
HAP	2 (4.1)	1 (5.2)	7 (11.5)	8 (12.3)	
VAP	0 (0)	0 (0)	1 (1.6)	0 (0)	
UTI	1 (2.0)	3 (14.3)	8 (13.1)	12 (18.5)	
IAI	35 (71.5)	15 (71.4)	33 (54.1)	26 (40)	
BSI	0 (0)	0 (0)	0	6 (9.2)	
Underlying conditions, number (percentage)					0.049
COPD	5 (10.2)	0 (0)	3 (4.9)	6 (9.2)	
DM2	9 (18.4)	2 (9.5)	11 (18.0)	10 (15.4)	
Heart failure	4 (8.2)	2 (9.5)	8 (13.1)	6 (9.2)	
CRD	0 (0)	0 (0)	2 (3.3)	7 (10.8)	
Malignancy	16 (32.6)	6 (28.6)	21 (32.3)	26 (40.0)	

APACHE II, Acute Physiology and Chronic Health Evaluation II; BSI, bloodstream infection; CAP, community-acquired pneumonia; COPD, chronic obstructive pulmonary disorder; CRD, chronic renal disease; DM2, diabetes mellitus type 2; HAP, hospital-acquired pneumonia; IAI, intrabdominal infection; SD, standard deviation; suPAR, soluble urokinase plasminogen activator receptor; UTI, urinary tract infection; VAP, ventilator-associated pneumonia.

(a) Age. The Swedish cohort involved younger patients. Their mean age was 61.2 years with a standard deviation of 15.8 years (*P *= 0.001 compared with the Greek cohort).

(b) Disease severity. Among the 196 patients, two patients (1.0%) had sepsis, 43 patients (21.9%) had severe sepsis, and 151 patients (77.1%) had septic shock (*P *< 0.0001 compared with the Greek study cohort). Consequently, the APACHE II scores were significantly higher in the Swedish study cohort (*P *< 0.0001).

(c) Type of hospitalization. All but five Swedish patients were admitted to an ICU.

(d) Underlying infection causes of sepsis. Among the 196 patients, intra-abdominal infections predominated (*P *< 0.0001 compared with the Greek cohort). The median length of stay in the ICU was 6 days (range of 3 to 12 days).

Similar to the findings in the Greek cohort, suPAR levels in the Swedish cohort were significantly higher in non-survivors; the median levels were 15.72 ng/mL (range of 6.45 to 46.60 ng/mL) in non-survivors and 10.09 ng/mL (range of 1.10 to 42.22 ng/mL) in survivors (*P *< 0.0001).

Respective mortalities of the Swedish patients in relation to the four levels of strata (i), (ii), (iii), and (iv) of the prognostication score were 2.0%, 28.6%, 21.3%, and 33.8% (*P *< 0.0001 within the four levels). The high mortality in stratum (ii) may be due to small number of patients in this group (n = 21) and chance variations in mortality. Apart from that aberration, the findings validate those from the larger Greek study, even though patients in the two groups are very different (Table 5).

## Discussion

A simple risk stratification system was developed for patients with sepsis by taking into consideration APACHE II score and the serum biomarker suPAR. The Greek and Swedish cohorts of patients used to generate and confirm this risk stratification system are indicative of the local epidemiology of sepsis in Greece and Sweden as defined after comparisons with published series of Greek and Swedish patients [20-22]. APACHE II score is the gold standard for risk assessment of critically ill patients [2]. However, the score is known to provide misleading values in certain patients, such as disproportionately high scores in older patients with chronic organ failure or in patients in a coma. This translates into a clinical reality in which the negative predictive value of APACHE II score to forecast death is not as satisfactory as clinicians would expect. We propose to improve prognostication by APACHE II score through the inclusion of stratification by suPAR, a serum/plasma biomarker that is easily performed on-site and provides information within 1 hour [23].

APACHE II scores and suPAR levels are used to create four independent stratification levels of risk for unfavorable outcome. Patients initially considered at low risk on the basis on the APACHE II score are thus stratified into two populations: those who are truly at low risk for death and have both low APACHE II scores and low serum suPAR and those who are falsely considered at low risk of death and who have low APACHE II scores but in whom elevated serum suPAR marks a higher risk of death. The validity of this novel approach of risk assessment is confirmed in a second patient cohort. The confirmation is valuable for two main reasons: the patients come from a different geographic region with a different health-care system, and stratification by suPAR proves its value even when the cohort used for confirmation involves patients who are younger and more severely ill and have different types of infection as causes of sepsis. Results are similar in the two cohorts; patients with a low APACHE II score but elevated suPAR are at considerable risk of dying. Conversely, patients with a high APACHE II score but a low suPAR may still risk dying, but the risk is significantly lower than in patients who score high on both APACHE II and suPAR.

suPAR remains stable in the systemic circulation in both survivors and non-survivors within the first 10 days of the disease course. This is consistent with a recent study of 271 critically ill patients [7] and is clearly of major importance for risk assessment. Given the stability of suPAR over the disease course, the validity of the developed prognostication score remains constant even if suPAR is not measured within the first day of diagnosis. The findings are comparable to those of other diseases − like chronic HIV-1 infection [3,4] and tuberculosis [24] − in which suPAR prognosticates an unfavorable outcome.

Sepsis causes high mortality, exceeding 30% for patients with severe sepsis and rising to over 50% for patients with septic shock. Figures have improved in the last decade, probably thanks to improved early recognition of sepsis, but effective new therapies are lacking. As a consequence, improving outcome is a multifaceted task. One crucial measure is early and correct identification of high-risk patients in need of early intervention [25]. This study proposes one way of doing that. The proposed score allows stratification of patients with sepsis at four strata of risk for unfavorable outcome on the basis of APACHE II score and serum suPAR. Its negative predictive value of 94.5% makes the score useful in clinical practice. It may also guide decision-making in countries with a shortage of ICU beds. In those settings, selection of patients in real need of intensive care should rely not only on clinical judgment but also on the proposed score. Also, in a situation in which ICU beds are not available, this new stratification indicates which patients in the general ward should be intensively monitored. This applies even to patients who have uncomplicated sepsis but whose mortality remains between 5% and 10%, meaning that some will deteriorate over time. The use of the biomarker suPAR, which remains stably elevated for 10 days, in combination with APACHE II score may help to offer intensive care management early in these patients.

The use of scoring systems to guide decision-making in sepsis has been thoroughly criticized. The most recent example is the administration of recombinant human activated protein C licensed for patients with an APACHE II score of at least 25. It is proposed that guidance of sepsis therapy by biomarkers may easily fail because available scoring systems (APACHE II, in particular) manage to recognize either low-risk patients or very-high-risk patients but not the patients in between these two extremes [26]. The proposed risk stratification score fulfills this need because it recognizes not only patients lying at one of the two extremes - strata (i) and (iv) - but also patients lying in between, namely patients with an APACHE II score of less than 17 and suPAR of at least 12 ng/mL or patients with an APACHE II score of at least 17 and suPAR of less than 12 ng/mL, who belong to strata (iii) and (iv), respectively.

## Conclusions

A novel prediction rule with four levels of risk in sepsis is proposed. The rule is based on a composite risk stratification that uses APACHE II score and serum levels of suPAR. The value of this rule is based on the good risk assessment of patients not detected by APACHE II. Effective prognostication is confirmed by an independent cohort.

## Key messages

• New risk stratification is introduced for sepsis on the basis of APACHE II and the novel biomarker suPAR. This stratification allows early identification of patients at real risk for death, even when APACHE II score is low. The negative predictive value of this score is 94.5%.

• The validity of this score is confirmed by an independent cohort of patients from Sweden.

## Abbreviations

APACHE II: Acute Physiology and Chronic Health Evaluation II; AUC: area under the curve; BAL: bronchoalveolar lavage; CAP: community-acquired pneumonia; CI: confidence interval; CRF: Case Report Form; ICU: intensive care unit; OR: odds ratio; ROC: receiver operator curve; suPAR: soluble urokinase plasminogen activator receptor; TBS: tracheobronchial secretion.

## Competing interests

The authors declare that they have no competing interests.

## Authors' contributions

EJG-B designed the study, performed statistical analysis, and wrote the manuscript. AN-T, AL, JS-C, and C-JT collected and analyzed data from the Swedish cohort and helped to draft the manuscript. VM, AS, IT, ID, MR, GA, AAn, EA, MC, CK, IKo, KK, GK, MK, IKr, KL, KM, AMa, AMe, AP, and CR collected clinical data and helped to draft the manuscript. MM monitored the study and helped to draft the manuscript. MG performed lab measurements and helped to draft the manuscript. CG, AAr, and GD participated in the study design and the analysis of data and helped to draft the manuscript. All authors read and approved the final manuscript.
